# Shape-Memory Effect Triggered by π–π Interactions in a Flexible Terpyridine Metal–Organic Framework

**DOI:** 10.1021/acsmaterialslett.3c00068

**Published:** 2023-03-23

**Authors:** Kornel Roztocki, Wiktoria Gromelska, Filip Formalik, Alessia Giordana, Luca Andreo, Ghodrat Mahmoudi, Volodymyr Bon, Stefan Kaskel, Leonard J. Barbour, Agnieszka Janiak, Emanuele Priola

**Affiliations:** † Faculty of Chemistry, Adam Mickiewicz University, Uniwersytetu Poznańskiego 8, 61-614 Poznań, Poland; ‡ Department of Micro, Nano, and Bioprocess Engineering, Faculty of Chemistry, Wroclaw University of Science and Technology, 50-370 Wroclaw, Poland; § Department of Chemical and Biological Engineering, Northwestern University, Evanston, Illinois 60208, United States; ⊥ Dipartimento di Chimica, Università degli Studi di Torino, Via Pietro Giuria 7, 10125, Torino, Italy; ¶ Department of Chemistry, Faculty of Science, University of Maragheh, P.O. Box 55136-83111, Maragheh 83111-55181, Iran; ∥ Chair of Inorganic Chemistry, Technische Universität Dresden, Bergstrasse 66, 01062 Dresden, Germany; ◆ Department of Chemistry and Polymer Science, University of Stellenbosch, Private Bag X1, Matieland 7602, South Africa

## Abstract

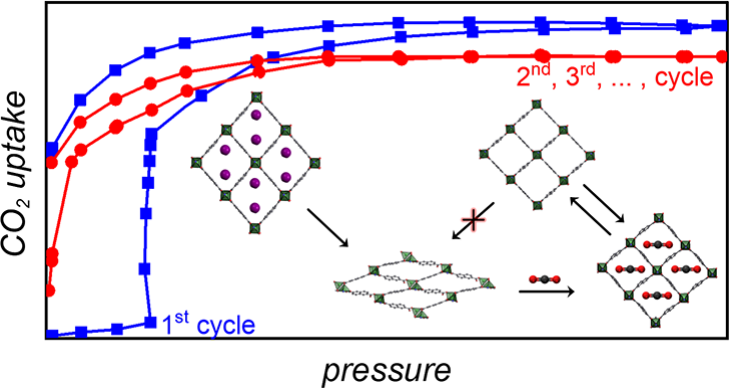

Shape-memory polymers and alloys are adaptable materials capable of reversing from a deformed, metastable phase to an energetically favored original phase in response to external stimuli. In the context of metal–organic frameworks, the term shape-memory is defined as the property of a switchable framework to stabilize the reopened pore phase after the first switching transition. Herein we describe a novel flexible terpyridine MOF which, upon desolvation, transforms into a nonporous structure that reopens into a shape-memory phase when exposed to CO_2_ at 195 K. Based on comprehensive *in situ* experimental studies (SC-XRD and PXRD) and DFT energetic considerations combined with literature reports, we recommend dividing shape-memory MOFs into two categories, viz responsive and nonresponsive, depending on the transformability of the gas-free reopened pore phase into the collapsed phase. Furthermore, considering the methodological gap in discovering and understanding shape-memory porous materials, we emphasize the importance of multicycle physisorption experiments for dynamic open framework materials, including metal–organic and covalent organic frameworks.

Flexible metal–organic frameworks (MOFs) are porous coordination polymers that undergo considerable structural transformation upon desolvation, i.e., the process of removing the guest molecules from the as-synthesized material.^1−4^ Desolvation of gating MOFs usually causes the open structure (*op*) to collapse, leading to a less porous or nonporous crystalline solid designated as the closed phase (*cp*). Exposing *cp* to gas molecules reconstructs the porosity (*cp* → *op*), which is abruptly filled with guest through an adsorption process.^5^ The gas-induced reopening exhibits novel phenomena,^6−15^ inter alia the shape-memory effect,^16−20^ not observed for classical rigid adsorbents such as zeolites, porous carbons, and mesoporous silica.^21^ The switchable nature of flexible MOFs manifests as singularities^22,23^ in isothermal adsorption profiles, which cannot be assigned to any of the isotherm shapes classified by IUPAC.^24^ As an example, MIL-53 shows breathing behavior, with two-step CO_2_ or CH_4_ adsorption profiles.^25^ On the other hand, ELM-11 exhibits one-step gating adsorption, which originates from collective layers separation during CO_2_, N_2_, or Ar physisorption,^26^ while the structure of CoBDP changes several times, as reflected in its multistep N_2_ adsorption profile.^27^

Although gating MOFs switch to nonporous structures upon gas desorption,^23^ to the best of our knowledge, five flexible frameworks are known to exhibit permanent porosity even in the absence of gas molecules.^16−20^ These flexible MOFs are referred to as shape-memory MOFs since they do not change structure after the first switching transition despite their *cp* structure being energetically favored. This acquired rigidity is evident as type I adsorption isotherms for the second and subsequent adsorption cycles. The first shape-memory^16^ MOF, Cu_2_(bdc)_2_(bpy), reported by the Kitagawa group, exists in the metastable open form for a sample that contains crystals in a well-defined size regime. However, heating causes reversion to the closed phase. Other examples of shape-memory MOFs include two porous *pcu* frameworks,^17,18^ X-pcu-3-Zn-3i and X-pcu-1-Zn-3i, described by Zaworotko and co-workers, as well as magnesium frameworks,^19^ CPM-107, published by the Bu group. The shape-memory phases of these MOFs may be easily regenerated to the closed forms by treating them with high temperature and low pressure, or via repeating the activation process. On the other hand, the Farha group has shown that the shape-memory phase of Cu(4-PyC)_2_ is stable and the applied stimulus was not able to reverse it to the closed phase.^20^ Considering the above-mentioned examples, we recommend the division of shape-memory MOFs into two subgroups: (i) responsive, in which the shape-memory phase may be easily reversed to the closed phase; and (ii) nonresponsive, in which external stimuli do not transform the reopened MOF to the closed phase.

Herein, we report a novel flexible terpyridine MOF which, upon desolvation, transforms into the closed phase, and then to the open shape-memory phase (Figure 1) after the first CO_2_ (195 K) adsorption–desorption cycle. Detailed insight into the mechanisms of the structural transformations was obtained by applying sophisticated experimental and theoretical methods. Utilizing single-crystal diffraction (SC-XRD) analysis, we determined five guest-dependent single-crystal phases, including the CO_2_-loaded shape-memory phase, while complementary cycling *in situ* powder X-ray diffraction (PXRD) measurements indicated the ability to maintain permanent porosity during repeated adsorption and desorption stress. Structural analysis confirms that the reopened phase is stabilized by intermolecular π–π interactions between the terpyridine linkers. Furthermore, theoretical energy considerations indicate that the obtained shape-memory phase is thermodynamically unstable at low temperature.

**Figure 1:**
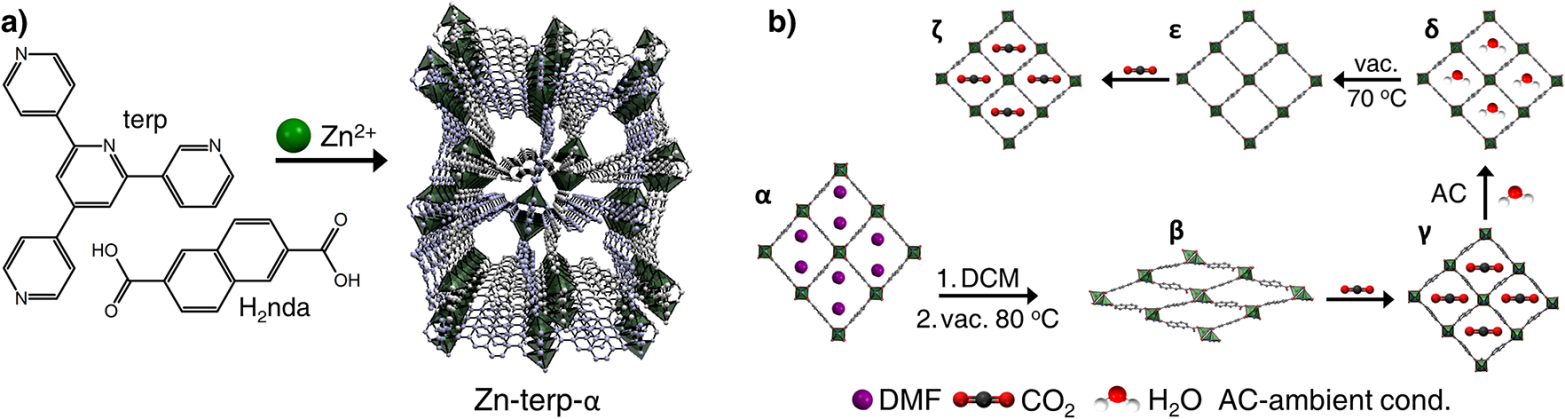
a) Synthetic route and structural features of as-synthesized Zn-terp-α along with b) schematic illustration of the phase transformation triggered by several subsequent stimuli; for clarity, one subframework representing the doubly interpenetrated Zn-terp-x: x = α – [Zn_2_(nda)_2_(terp)]·2DMF, β – [Zn_2_(nda)_2_(terp)], γ – [Zn_2_(nda)_2_(terp)]·3CO_2_, δ – [Zn_2_(nda)_2_(terp)]·2H_2_O and ε – [Zn_2_(nda)_2_(terp)]; ζ – [Zn_2_(nda)_2_(terp)]·CO_2_. nda^2–^ – 2,6-naphthalenedicarboxylate, terp – 6′-(pyridin-4-yl)-3,2′:4′,4″-terpyridine, DCM – dichloromethane. Only the γ-phase was not structurally characterized by SC-XRD analysis. Before exposure to ambient conditions, the γ-phase was degassed at room temperature and low pressure.

Reaction of the N-donor linker 6′-(pyridin-4-yl)-3,2′:4′,4″-terpyridine (terp), 2,6-naphthalene dicarboxylic acid (H_2_nda) and zinc nitrate in an N,N′-dimethylformamide (DMF) and ethanol mixture at 80 °C yielded colorless, block-shaped crystals, [Zn_2_(nda)_2_(terp)]·2DMF (Zn-terp-α; Figures 1, 2a, S1, S2). SC-XRD analysis revealed that the material crystallizes in the triclinic space group P1̅ (Table S1). Zinc cations form binuclear “paddlewheel” molecular building blocks linked equatorially by the *μ*_*4*_*-κ*^*1*^*κ*^*1*^*κ*^*1*^*κ*^*1*^ anions nda^2–^ to Zn_2_(nda)_2_, resulting in a square lattice (*sql*) network. The terp linkers further connect the layers into a 3D network exhibiting the primitive cubic (*pcu*) topology. Interestingly, the terp linker contains three outer pyridine rings and may be considered as a *μ*_*3*_*-κ*^*1*^*κ*^*1*^*κ*^*1*^ linker. Nevertheless, only two of them, via nitrogen atoms (*μ*_*2*_*-κ*^*1*^*κ*^*1*^), are axially bound to the paddlewheel unit, while the third one does not coordinate. Zn-terp-α consists of 2-fold interpenetrated networks with a two-dimensional pore system and 35% void space occupied by DMF (probe radius = 1.2 Å).

**Figure 2:**
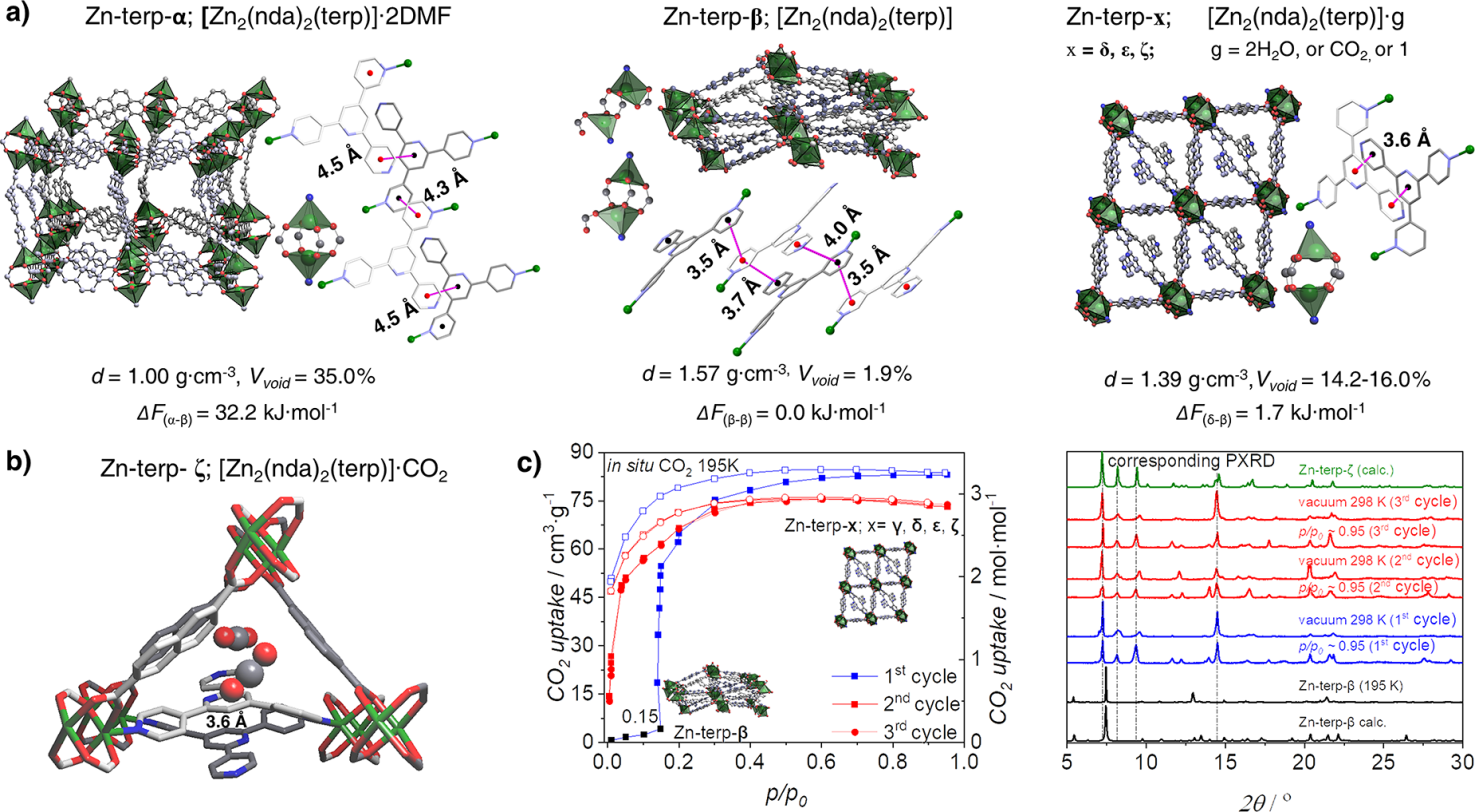
a) Evolution of guest-dependent crystalline phases of Zn-terp-α elucidated by *ex situ* and *in situ* SC-XRD: doubly interpenetrated networks, coordination environments of dizinc unit, and π–π interactions of terp linkers. (For more details, see Table S2, Figure S3.) H atoms are omitted for clarity. C – gray, N – blue, O – red, Zn – green. *d*: calculated density excluding guest molecules (see Table S1). Void fractions (*V*_void_) were estimated using Mercury (probe radius = 1.2 Å). Δ*F*: relative free energies of structures at 1 K calculated using DFT (see Table S3). b) CO_2_ binding sites involving nda^2–^ anions and terp linkers resolved from *in situ* SC-XRD data collected during CO_2_ adsorption at 296 K. c) Three CO_2_ adsorption (full symbols) and desorption (open symbols) cycles at 195 K juxtaposed with calculated and experimental PXRD patterns (λ = 1.540599 Å; for more details, see Figure S4).

Removal of the solvent from the α-phase triggers significant structural changes (Figure 2a), e.g. the unit cell volume of the new nonporous β-phase ([Zn_2_(nda)_2_(terp)]), is reduced by 34%, while the void fraction decreases from 35.0% to 1.9%. Detailed structural analysis of the terp aromatic rings reveals a guest-dependent evolution of π–π interactions (Table S2). In the α-phase, terpyridine linkers form a chain motif stabilized by intermolecular π–π interactions in which the shortest centroid-to-centroid distances are 4.346(5) Å. Although the α→β transformation does not affect the number of interactions, it changes the share of individual rings in these types of interactions. Thus, in the α-phase π–π interactions occur between two outer rings and the outer and central rings of two adjacent terpyridine linkers, while in the β-phase, these interactions occur between three adjacent linkers and involve only the outer rings (Figure 2a). Consequently, the centroid-to-centroid distances are reduced to 3.516(5) Å. Zn-terp-α contains highly symmetrical paddlewheel units (*pwu*; ∠ *nda-pwu-nda* = 87.74° to 92.47°) with terp linkers aligned in-plane relative to each other. The contraction process disassembles the paddlewheel units, thus forming two different secondary building blocks in each subframework.

Isothermal physisorption of CO_2_ at 195 K (Figure 2b and Figure S5) reveals that the collapsed β-phase does not interact with the adsorbate until the pressure reaches *p*/*p*_0_ = 0.15, after which the MOF immediately transforms into an unknown open γ-phase [Zn_2_(nda)_2_(terp)]·3CO_2_. Owing to low symmetry of the system (P1̅) and cracking of the majority of the crystals during the adsorption process, we were not able to accurately determine the structure of this phase using either *in situ* PXRD or *in situ* SC-XRD data. However, using SC-XRD, we determined structures of three analogous phases including: *i*) Zn-terp-δ [Zn_2_(nda)_2_(terp)]·2H_2_O, the phase that traps two water molecules from the air after desorption of CO_2_; *ii*) Zn-terp-ε, the water-free phase; and *iii*) the Zn-terp-ζ phase containing CO_2_ molecules that are enclosed by nda^2–^ and terp linkers (Figure 2 and Figure S6).

Stabilization of the shape-memory phases (δ, ε, ζ) originates from mutual positions of two terp linkers interacting by a pair of intermolecular π–π interactions, characterized by centroid-to-centroid distances of 3.593(5)–3.598(6) Å (Table S2). Thus, the crystal structures of the δ-, ε-, ζ-phases have comparable cell volumes, densities, connectivity, and free void fractions (Figure 2, Figure S8, Table S1). Furthermore, the adsorption partially reverses the structural changes caused by desolvation, e.g. the rebuilt paddlewheel units are deformed (∠ *nda-pwu-nda* in the range 84.80°–97.62°). Two subsequent adsorption–desorption cycles monitored by *in situ* PXRD and SC-XRD experiments indicate that the shape-memory phase behaves as a rigid solid, albeit with slight changes in PXRD patterns during the repeated adsorption–desorption arising from crystallographically defined adsorbate positions in the framework (Figure 2b and Figure S6).

We applied DFT for the first time to understand the observed phenomenon. For this purpose, we utilized guest-free Zn-terp-δ as the shape-memory phase in the temperature-dependent free energy DFT calculations (Figure 3). Analogous calculations were carried out for the pristine α-phase and the closed β-phase. Comparison of results indicates that the pristine α-phase is thermodynamically unstable over the entire temperature range, which explains why it can be easily transformed into the closed β-phase during desolvation.

**Figure 3:**
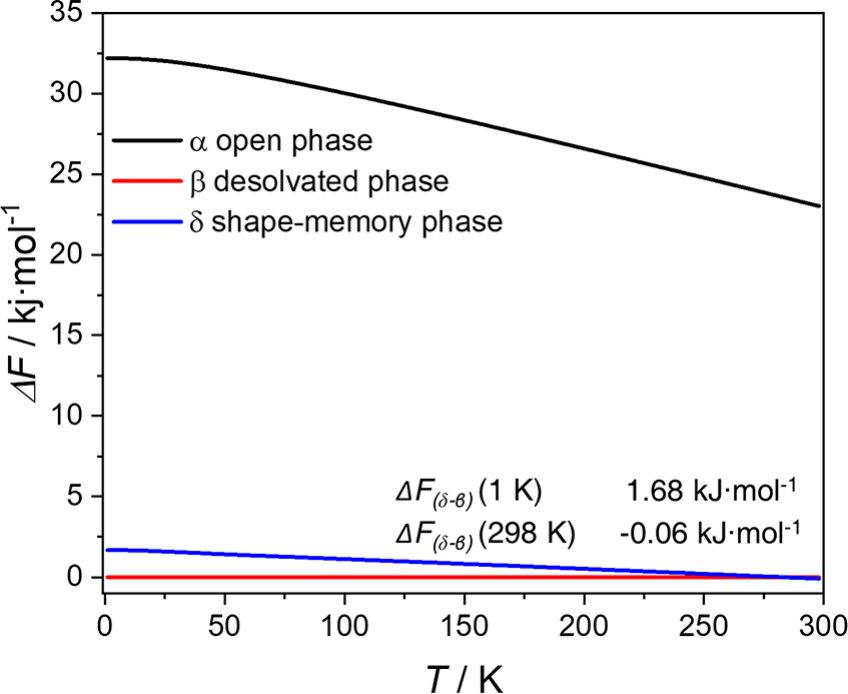
Calculated temperature-dependent relative free energy of α-, β-, δ-phases with free energy of the β-phase as a reference. Energy units are given per mole of Zn paddlewheel.

Despite applying a plethora of stimuli to the δ-shape-memory phase, e.g. repeated activation, we did not observe transition from the δ-phase to the closed β-phase or open α-phase (Figure S9). Our simulations indicate that the δ- and β-phases have comparable energies at room temperature, although they do not include the energy barrier, which must be sufficiently high to prevent the δ→β phase transformation, possibly due to bond breaking within Zn paddlewheel. Owing to the technical complexity of elucidating the path of transition between the discussed phases, theoretical calculations of the free energy barriers are beyond the scope of this work and will be the subject of further detailed experimental and theoretical investigation in the future. Although the free energy of the δ shape-memory phase is higher than that of the β-phase (i.e., δ is less stable), the difference is so small that we predict that both phases may coexist at low temperature.

We have combined structural analysis (SC-XRD and PXRD) with DFT methodology to comprehensively investigate a flexible terpyridine MOF, which transforms into a stable phase during the first CO_2_-induced transition. The observed phenomenon originates from the evolution of intermolecular π–π interactions between the terpyridine linkers, while the adsorbed CO_2_ molecules are located in pockets formed by naphthalenedicarboxylates and terpyridine linkers. Furthermore, our DFT approach is the first example of a theoretical methodology describing shape-memory MOFs and thus paves the way for further developments in this field.

Considering our findings and those reported in the literature, we propose systematizing the terminology relevant to shape-memory in MOFs, dividing them into the two subgroups responsive and nonresponsive. Moreover, we recognize the methodological gap in the discovery and understanding of this undeveloped phenomenon. Accordingly, we emphasize the essential role of multicycle physisorption experiments as well as the development of theoretical tools for further discoveries and understanding of shape-memory porous materials, including flexible noncovalent-, covalent- and metal–organic framework.

## Supporting Information Available

PXRD patterns, crystal structure drawings, synthetic procedures, and additional experimental data (PDF)
X-ray crystal structure for Zn-terp-α (CIF)
X-ray crystal structure for Zn-terp-β (CIF)
X-ray crystal structure for Zn-terp-δ (CIF)
X-ray crystal structure for Zn-terp-ε (CIF)
X-ray crystal structure for Zn-terp-ζ (CIF)

## Supplementary Material

tz3c00068_si_001.pdf

tz3c00068_si_002.cif

tz3c00068_si_003.cif

tz3c00068_si_004.cif

tz3c00068_si_005.cif

tz3c00068_si_006.cif

## Notes

The preprint version of this work is available on *ChemRxiv*: Roztocki, Kornel; Gromelska, Wiktoria; Formalik, Filip; Giordana, Alessia; Andreo, Luca; Mahmoudi, Ghodrat; Bon, Volodymyr; Kaskel, Stefan; Barbour, Leonard; Janiak, Agnieszka; Priola, Emanuele; Shape-memory effect triggered by π–π interactions in a flexible terpyridine metal–organic framework, 2022, *ChemRxiv*. Cambridge: Cambridge Open Engage. https://chemrxiv.org/engage/chemrxiv/article-details/636b522780c9bf06d18f501e (accessed March 16, 2023).
