# Supplementary material for: Shape-Memory Effect Triggered by π–π Interactions in a Flexible Terpyridine Metal–Organic Framework
Source: ACS Mater Lett. 2023 Mar 23;5(4):1256–60. doi: 10.1021/acsmaterialslett.3c00068 (PMC10074452; doi:10.1021/acsmaterialslett.3c00068)
Supplement: Supplementary file 1 — tz3c00068_si_001.pdf [file tz3c00068_si_001.pdf]

SUPPLEMENTARY INFORMATION FILE ACCOMPANYING:

Shape-memory effect triggered by π-π interactions in a flexible terpyridine metal-organic framework

Kornel Roztocki[a], Wiktoria Gromelska[a], Filip Formalik[b,c], Alessia Giordana[d], Luca Andreo[d], Ghodrat Mahmoudi*[e], Volodymyr Bon[f], Stefan Kaskel[f], Leonard J. Barbour[g], Agnieszka Janiak[a] and Emanuele Priola[d]*

[a] Faculty of Chemistry, Adam Mickiewicz University, Uniwersytetu Poznańskiego 8, 61-614 Poznań, Poland
[b] Department of Micro, Nano, and Bioprocess Engineering, Faculty of Chemistry, Wroclaw University of Science and Technology, 50-370 Wroclaw, Poland
[c] Department of Chemical and Biological Engineering, Northwestern University, Evanston, Illinois, 60208, United States
[d] Dipartimento di Chimica, Università degli Studi di Torino, Via Pietro Giuria 7, 10125, Torino, Italy
[e] Department of Chemistry, Faculty of Science, University of Maragheh, P.O. Box 55136-83111, Maragheh, Iran
[f] Chair of Inorganic Chemistry, Technische Universität Dresden, Bergstrasse 66, 01062 Dresden, Germany
[g] Department of Chemistry and Polymer Science, University of Stellenbosch, Private Bag X1, Matirland 7602, South Africa

Table of Contents

Experimental and computational procedures 2

Figures 6

Tables 14

Figures

Figure S1 Confirmation of the purity of the pristine bulk phase: experimental and calculated PXRD patterns of the as-synthesized materials Zn-terp-α (left) juxtaposed with optical image of it (right). 6
Figure S2 X-ray crystal structures of pristine framework Zn-terp-α**: a)** coordination environments of di-zinc paddle wheel unit. **b)** Representation of 2D layers $[Zn(nda)_2]_n$. **c)** single subnetwork. **d)** doubly interpenetrated networks, **e)** Contact surface and fraction of voids (V_{voids}) were calculated with Mercury software by using a probe molecule with a radius of 1.2 Å (views along the a axes). **f)** structural futures of Zn-terp-α.H atoms were omitted for clarity. Color codes: Zn-green, O-red, N -blue, C-grey, voids – yellow. 6
Figure S3 Evolution of di-zinc unit of Zn-terp-α and π-π interactions of terp linkers observed during constitutive stimuli, desolvation and CO_2 adsorption at 296 K. 7
Figure S4 Three CO_2 adsorption (full symbols) and desorption (open symbols) cycles at 195 K juxtaposed with corresponding in situ PXRD patterns (λ = 1.540599 Å), measured in the almost all points of the presented isotherms. 8
Figure S5 First indication of shape-memory effect: adsorption isotherms of CO_2 (195 K) and N_2 (77 K) for activated (exchange DMF to DMC, fine vacuum at 80 °C) Zn-terp-β $[Zn_2(nda)_2(terp)]_n$. Full symbols – adsorption; open symbols – desorption. 9
Figure S6 CO_2 binding sites resolved from in situ SC-XRD data collecting during the CO_2 adsorption at 296 K. The solvent accessible volume shown as green Connolly surfaces was generated with a probe radius of 1.5 Å. 9
Figure S7 An overlay (left) of the shape-memory MOF structures under reduced (black) and 10 bar CO_2 (magenta) pressure juxtaposed with the unit cell volume changes during the 2nd adsorption cycle (see Fig. S4). 10
Figure S8 Experimental PXRD patterns of Zn-terp-γ, Zn-terp-δ, Zn-terp-ε and Zn-terp-ζ showing that all structure are analogous (left). Pore size distribution (PSD) for Zn-terp-δ, Zn-terp-ε and Zn-terp-ζ juxtaposed

with unit cell parameters and theoretical pore volume calculated with Zeo++ software (right); all calculations were made with guest molecules excluded. 10
Figure S9 Comparison of the the PXRD patterns of as-synthesised δ-phase with those after soaking for 24h in DCM and DMF. Immersion of the α-phase in DMF significantly reduces crystallinity of the sample, while DCM has not impact on the sample. 11
Figure S10 Thermal stability (left) of Zn-terp-α $[Zn_2(nda)_2(terp)\cdot 2DMF]_n$ indicating structural transformation into Zn-terp-β $[Zn_2(nda)_2(terp)]_n$ juxtaposed with calculated temperature-dependent relative free energy (right) of the α- ,β- ,δ- phases with free energy of β as a reference; Energy unit is given per mole of Zn paddle-wheel. 11
Figure S11 Experimental and calculated PXRD patterns of the desolvated materials Zn-terp-β (left) juxtaposed with optical image of it (right). AC - ambient conditions. 12
Figure S12 (top)^{1}H NMR spectrum of terp in DMSO-d6 and (down) ^{13}C NMR spectrum of terp in DMSO-d6. 13
Figure S13 HSQC spectrum of terp. 13

Tables

Table S1 Crystallographic data for Zn-terp-α, β, δ, ε and ζ. 14
Table S2 Evolution of π-π interactions observed in different phases of Zn-terp-x. 15
Table S3 Lattice parameters and relative energies of selected structures. 15
Table S4 Relative free energies for α and β phases with free energy of δ as a reference. Energies given per 1 Zn paddlewheel. 15

Experimental and computational procedures

General Remarks. All reagents and solvents were of analytical grade (Sigma Aldrich, Merck, FluoroChem) and were used without further purification. Unless otherwise stated, all manipulations were done under ambient conditions.

Synthesis of 6'-(pyridin-4-yl)-3,2':4',4''-terpyridine (terp): The 4-acetylpyridine (2.05 g, 0.017 mol) was added to a solution of the 3-pyridine carboxylaldehyde (0.92 g, 0.009 mol) in ethanol (50 mL), then granulated KOH (1.18 g, 0.021 mol) and NH_3 (25% solution in water, 4 mL) were added to the mixture. The reaction mixture was stirred at r.t for 4 h. The resulting crystals were filtered off, washed with water, petroleum ether and recrystallized from a mixture of alcohol-$CHCl_3$. This procedure is a modified method from the paper[1].

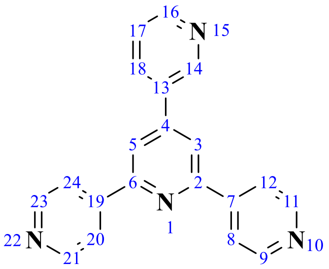

Scheme S1 Structure of 6'-(pyridin-4-yl)-3,2':4',4''-terpyridine linker (terp).

Yield 16%, white crystals. m.p. = 233-235 °C. NMR ^{1}H (DMSO-d_6, 300.1 MHz): δ 9.25 (1H, d, J = 2.3 Hz, H-14), 8.79-8.77 (4H, m, H-9, 11, 21, 23), 8.75-8.53 (1H, m, H-16), 8.46-8.42 (3H, m, H-3, 5, 18), 8.29-8.27 (4H, m, H-8, 12, 21, 24), 7.62-7.57 (1H, m, H-17) ppm. NMR ^{13}C (DMSO-d_6, 75.5 MHz): δ 155.4, 150.9, 150.8, 148.8, 148.2, 145.9, 135.3, 133.3, 124.2, 121.6, 119.5 ppm. IR ν_{max}/cm^{-1} (KBr): 3058, 3017, 1615, 1573, 1529, 1515, 1178. Elemental Analysis calcd. for $C_{20}H_{14}N_4$: C, 77.40; H, 4.55; N, 18.05; found C, 77.22; H, 4.46; N, 18.32.

[1] Smith, C. B.; Raston, C. L.; Sobolev, A. N., *Green Chem*. 2005, *7*, 650–654

Growing single crystals of Zn-terp-α, $[Zn_2(nda)_2(terp)\cdot 2DMF]_n$: To obtained crystals suitable to SC-XRD measurement, $Zn(NO_3)_2\cdot 6H_2O$ (13.8 mg, 0.046 mmol), 2,6-naphthalene dicarboxylic acid (10.0 mg, 0.046 mmol) and terpy-17 (14.3 mg, 0.046 mmol) were dissolved in a mixed solvent of DMF (9 ml) and EtOH (1 ml) and sonicated for few minutes to favour solubilisation. The vial containing the reaction mixture was sealed and placed in oven at 80°C for 46 hours and then the reaction system was slowly cooled to room temperature at a rate of 5°C/30 min. Colourless crystals were filtered to be collected, washed with DMF, and dried in air. Yield: 50% (based on $Zn(NO_3)_2\cdot 6H_2O$). The molecular formula is $Zn_2N_6O_{10}C_{50}H_{40}$ = $[Zn_2(nda)_2(terp)\cdot 2DMF]_n$, as derived from the structural refinement and elemental analysis. Elemental analysis (%): calc.: C 59.10, N 8.27, H 4.00; found: C 57.61, N 8.31, H 4.26.

Bulk synthesis of Zn-terp-α, $[Zn_2(nda)_2(terp)\cdot 2DMF]_n$: Using the same experimental condition but a 10 times more concentrated solution a crystalline powder with yield of 75% (based on $Zn(NO_3)_2\cdot 6H_2O$) was obtained.

Ambient single crystal X-ray diffraction. Single-crystal data for Zn-terp-α and Zn-terp-δ were collected on a Gemini R Ultra diffractometer, while for Zn-terp-β on Xcalibur diffractometer. Both instruments were equipped with a graphite-monochromatized Mo-Kα radiation ($\lambda = 0.71073$ Å) and the sample temperatures were controlled with an Oxford Instruments Cryojet Controller. Data collection, data reduction and multi-scan absorption collection were performed with the CrysAlisPro software [CrysAlis PRO 1.171.38.46 (Rigaku OD, 2015)]. Using the Olex^2 program[2], all structures were solved by direct methods using the SHELXS-14 program[3] and refined with full-matrix least-squares techniques on F^2 using the SHELXL-14 program[4]. All non-hydrogen atoms were refined anisotropically. Hydrogen atoms positions were calculated and refined riding on the corresponding bonded atoms. The SQUEEZE tool implemented in Olex2 program was used to subtract the contribution of disordered DMF solvent molecules from the diffraction data in Zn-terp-α ([Zn2(nda)2(terp)·2DMF]n). The estimated electron count is 360 in an accessible void volume of 2413Å3 and is correlated with approximately 2DMF. On the other hand, Zn-terp-δ phase ($[Zn_2(nda)_2(terp)\cdot 2H_2O]_n$) presents a water interacting with the free pyridyl nitrogen through hydrogen bond and a two position-disordered water, and both are clearly visible on the difference electron density map. In Zn-terp-ε and Zn-terp-ζ the nda^{2-} ligand is disordered over two symmetry correlated positions, with occupancy of 0.5 for each component of the disorder.

We have found that the Zn-terp-β form is only stable under argon. When the crystals are exposed to the atmospheric conditions they undergo single-crystal-to-single crystal transformation to the hydrated form. The process is spontaneous and X-ray intensity data recorded for the Zn-terp-β form also includes diffraction data for the partially hydrated form, which has slightly different unit cell parameters and geometry around Zn centers. Hence, high residual electron density as well as high R_{int}, R_1 and wR_2 are observed.

Single crystal X-ray diffraction under CO_2 pressure. The crystal structures determined from intensity data collected with the crystal under controlled atmospheres were carried out in an environmental gas cell developed in-house by prof. L.J. Barbour.. The gas cell consisted of a glass capillary attached to a stainless steel tube with an inlet/outlet valve through which the gas was introduced or the vacuum was applied. The system was sealed from the atmosphere. The crystal was glued to a glass fiber and placed in the glass capillary. The crystal selected for X-ray experiments was not pure single crystal (UB matrix fit with 56.08% of reflections), but the only one that survived the transformation to the shape-memory phase. The X-ray measurements were performed for both, the crystal under reduced (vacuum) and gas pressure (CO_2). Intensity data collected for Zn-terp-ε **(**$[Zn_2(nda)_2(terp)]_n$) and Zn-terp-ζ ($[Zn_2(nda)_2(terp)\cdot CO_2]_n$) was measured at 296 K on a SuperNova diffractometer equipped with Cu microfocus source (λ=1.54178 Å) and 135 mm Atlas CCD detector. Data reduction and analysis were carried out with the CrysAlisPro software (*v.* 171.41.123a, Rigaku OD, 2022). The

[2] O. V. Dolomanov, L. J. Bourhis, R. J. Gildea, J. A. K. Howard and H. Puschmann, *Journal of Applied Crystallography*, 2009, 42, 339-341.
[3] G. M. Sheldrick, *Acta Crystallogr. Sect. A*, 2008, 64, 112-122.
[4] G. M. Sheldrick, *Acta Crystallogr. Sect. A*, 2015, 71, 3-8.

crystal structures were solved by direct methods using the SHELXT[4] program and were refined by full matrix least squares on F^2 using the program SHELXL-2018[4] through the graphical interface X-Seed.[5] Non-hydrogen atoms were refined anisotropically except for those constituting the disordered model of the carbon dioxide which were refined isotropically. The hydrogen atoms bound to C atoms were placed at calculated positions and refined using a riding model, and their isotropic displacement parameters were given a value 20% higher than the isotropic equivalent for the atom to which the H atoms were attached.

In the crystal structure of Zn-terp-ζ($[Zn_2(nda)_2(terp)\cdot CO_2]_n$, the CO_2 molecule was identified in two regions: first (1) in close distance to the nda^{2-} linkers and second (2) near to terp linkers. In both cases, its position is disordered. In 1, the CO_2 displays a disorder where one of the two oxygen atoms is split into three positions with the final occupancy factors of 0.19, 0.18 and 0.18, and the remaining two atoms are common with fixed occupancies of 0.55 each. In 2, the CO_2 molecule were found to be disordered over four positions with the site occupancy factor of 0.18, 0.15, 0.07 and 0.05, summing up to the occupancy of 0.45. In all disorder models, the restraints for the 1,2- and 1,3-distances were applied.

The relevant crystal data collection and refinement parameters are listed in Table S1. CCDC 2218121, 2218122, 2218123, 2218254 and 2218254 contain the supplementary crystallographic data for this paper. These data can be obtained free of charge via www.ccdc.cam.ac.uk/data_request/cif, or by emailing data_request@ccdc.ca m.ac.uk , or by contacting The Cambridge Crystallographic Data Centre, 12 Union Road, Cambridge CB2 1EZ, UK; fax: +44 1223 336033.

NMR spectra were recorded on a Bruker ARX 300 spectrometer in DMSO-d6.

Elemental analysis was carried out by conventional microanalysis using a Perkin-Elmer 240C microanalyzer elemental analyser. The elemental analysis (C, H, N and S) were carried out using a Thermo FlashEA 1112 CHNS-O analyser.

Thermogravimetric analyses (TGA) were performed on a Thermogravimetric TA Instruments (TGA 2950hr) instrument at a heating rate of 10 °C min^{-1} in a temperature range of 25 – 700 °C (approx. sample weight of 8.5 mg). The measurements were carried out at atmospheric pressure under flowing air.

Powder X-ray diffraction (PXRD) patterns were measured at room temperature on a STOE STADI P diffractometer using Cu-Kα1 radiation (λ = 1.5405 Å) and a 2D detector (Mythen, Dectris). Measurements on the STOE were performed in transmission geometry using a rotating flatbed sample holder.

Isothermal adsorption analysis: nitrogen (77 K) and carbon dioxide (195 K) adsorption/desorption studies were performed on a BELSORP-max II adsorption apparatus (MicrotracBEL Corp.); 77 K was achieved by liquid nitrogen bath, 195 K was achieved by dry ice/isopropanol bath. Prior to the sorption measurements the samples of **1** were soaked a few times for 3-8 h in DCM and evacuated at 70 °C for 12-16 h.

***In situ* powder X-ray diffraction** In situ PXRD experiments on Zn-terp-α in parallel to CO_2 adsorption at 195 K were performed on Empyrean powder X-ray diffractometer (ω - 2θ goniometer, K-alpha1 system) equipped with a home-built setup based on ARS DE-102 closed cycle helium cryostat (T = 30 – 300 ± 0.1 K) with customized *in situ* X-ray transparent adsorption cell. The cell was connected to the low-pressure port of the BELSORP-max volumetric adsorption instrument. The diffraction experiments were performed using ω - 2θ scans in transmission geometry in the range of 2θ = 3 – 90°. Parallel linear Cu-Kα1 irradiation, generated by hybrid 2xGe(220) monochromator, 4 mm mask, and primary divergence and secondary anti scatter slits with ¼° opening was used for data collection. Pixcel-3D Detector in 1D scanning mode (255 active channels) was used to measure the reflection intensities. A complete adsorption–desorption physisorption isotherm of carbon dioxide at 195 K was measured using 28 mg of sample, statically fixed in the flatbed sample holder, and PXRD patterns

[5] L. J. Barbour, *J. Appl. Cryst*. 2020, 53, 1141.

were recorded automatically after reaching adsorption equilibrium conditions (0.1% of pressure change within 300 s) at selected points of the isotherm. Sample was prepared in same way as for the isothermal adsorption analysis. On the same sample was measured three cycles. Between each cycle sample was evacuated for 2-3 hours under vacuum at 298 K. PXRD patterns, measured during the adsorption and desorption of CO_2 at 195 K during the 2nd cycle were indexed using DICVOL program, integrated in the FullProf.2k V.6.30. Further, the Le Bail fit was performed to refine the unit cell and profile parameters.

Computational Methodology
Before geometry optimization of the experimental structures, we removed disordered linkers with atoms with fractional occupancies as well as guest molecules (H_2O). DFT methodology was used for optimization, as implemented in VASP 5.4.4 with PAW potentials[6,7,8]. and PBE functional.[9] To properly account for long-range van der Waals interaction, we applied Grimme-type D3(BJ) dispersion correction[10], as it was previously proven that they are required to correctly predict the stability of flexible MOFs.[11] Only Γ-point was used to sample the Brillouin zone, due to the large unit cells of the structures. The energy cutoff was set to 600 eV and the SCF energy convergence criterion to 10^{-6} eV. We performed full geometry optimization (i.e., without volume and shape constraints) with a force convergence set to 10^{-2} eV/Å.

Phonons for the thermodynamical analysis were calculated using phonopy software[12,13]. We used only Γ point (1 **q**-point) of Brillouin zone due to relatively large unit cells of all phases and mesh grid of 6x6x6 for phonon determination. Helmholtz free energies were calculated using following equation:

$$F = E_{el} + \frac{1}{2}\sum_{\nu} \hbar\omega + k_b T \sum_{\nu} \ln\left(1 - e^{-\frac{\hbar\omega}{k_B T}}\right),$$

where E_{el} is a DFT energy and $\omega = 2\pi\nu$, (ν – frequency of mode). Second and third term of this equation are zero-point energy and vibrational free energy, respectively. All free energies presented in this work are relative free energies, with free energy of β phase (F_β) as a reference.

Geometry optimized structures have slightly lower volumes compared to experimental, except structure Zn-terp-δ. It is expected since additional factors not considered in simulations (e.g., temperature, pressure, or presence of guest molecules) may help to stabilize the experimental structure in larger volumes. Structures 3 and 4 (i.e., closed and shape-memory phase) are predicted to have the most stable among all considered (Table S2). Relative free energies for α and δ for selected temperatures are shown in Table S4.

[6]Kresse, G.; Hafner, J. *Phys. Rev. B* 1993, *47* (1), 558–561
[7]Kresse, G.; Furthmüller, J.*Computational Materials Science* 1996, 6 (1), 15–50
[8]Kresse, G.; Furthmüller, J. *Phys. Rev. B* 1996, 54 (16), 11169–11186
[9]Perdew, J. P.; Burke, K.; Ernzerhof, M. *Phys. Rev. Lett.* 1996, *77* (18), 3865–3868.
[10]Grimme, S.; Ehrlich, S.; Goerigk, L. *Journal of Computational Chemistry* 2011, *32* (7), 1456–1465.
[11]Wilmer, C. E.; Kim, K. C.; Snurr, R. Q. *J. Phys. Chem. Lett.* 2012, *3* (17), 2506–2511.
[12]A. Togo, I. Tanaka, *Scripta Materialia* 108 (2015) 1–5
[13] Atsushi Togo, Laurent Chaput, Isao Tanaka, and Gilles Hug *Phys. Rev. B* 81, 174301.

Figures

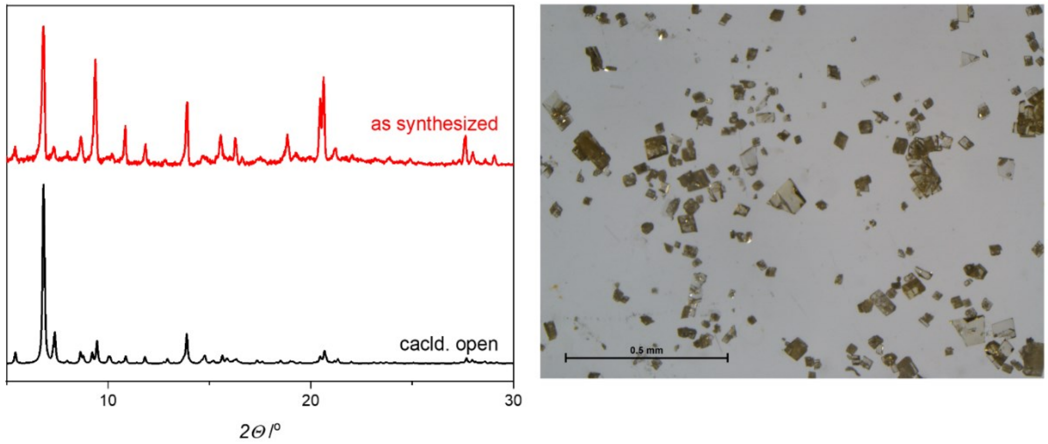

Figure S1 Confirmation of the purity of the pristine bulk phase: experimental and calculated PXRD patterns of the as-synthesized materials Zn-terp-α (left) juxtaposed with optical image of it (right).

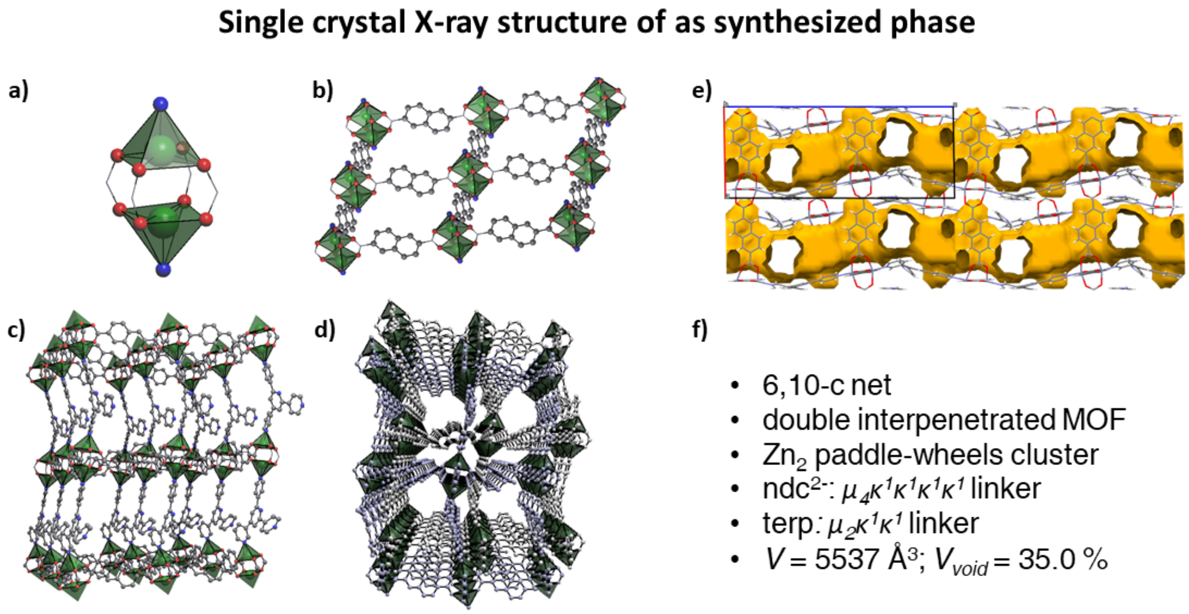

Figure S2 X-ray crystal structures of pristine framework Zn-terp-α**: a)** coordination environments of di-zinc paddle wheel unit. **b)** Representation of 2D layers $[Zn(nda)_2]_n$. **c)** single subnetwork. **d)** doubly interpenetrated networks, **e)** Contact surface and fraction of voids (V_{voids}) were calculated with Mercury software by using a probe molecule with a radius of 1.2 Å (views along the *a* axes). **f)** structural futures of Zn-terp-α.H atoms were omitted for clarity. Color codes: Zn-green, O-red, N -blue, C-grey, voids – yellow.

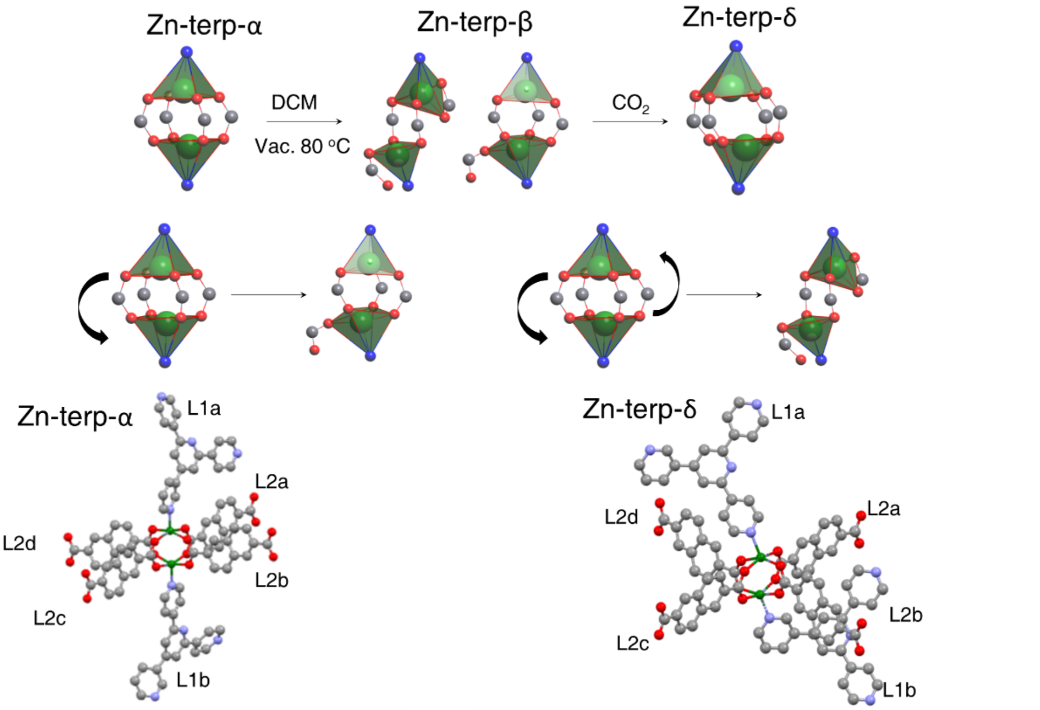

L1a-C-L2a	91,68	L1b-C-L2a	89,15	L2a-C-L2b	88,47
L1a-C-L2b	87,74	L1b-C-L2b	91,42	L2b-C-L2c	92,47
L1a-C-L2c	89,59	L1b-C-L2c	89,59	L2c-C-L2d	88,04
L1a-C-L2d	90,78	L1b-C-L2d	90,07	L2d-C-L2a	91,05

L1a-C-L2a	88,24	L1b-C-L2a	85,27	L2a-C-L2b	94,72
L1a-C-L2b	97,62	L1b-C-L2b	88,06	L2b-C-L2c	86,85
L1a-C-L2c	88,97	L1b-C-L2c	97,40	L2c-C-L2d	89,85
L1a-C-L2d	84,80	L1b-C-L2d	89,90	L2d-C-L2a	88,69

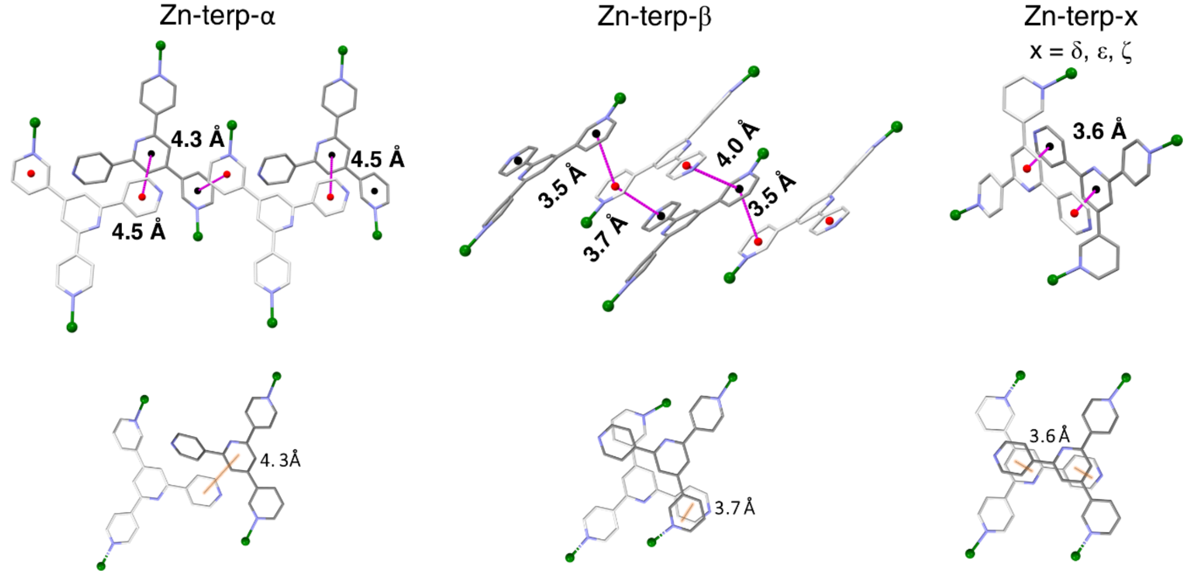

Figure S3 Evolution of di-zinc unit of Zn-terp-α and π-π interactions of terp linkers observed during constitutive stimuli, desolvation and CO_2 adsorption at 296 K.

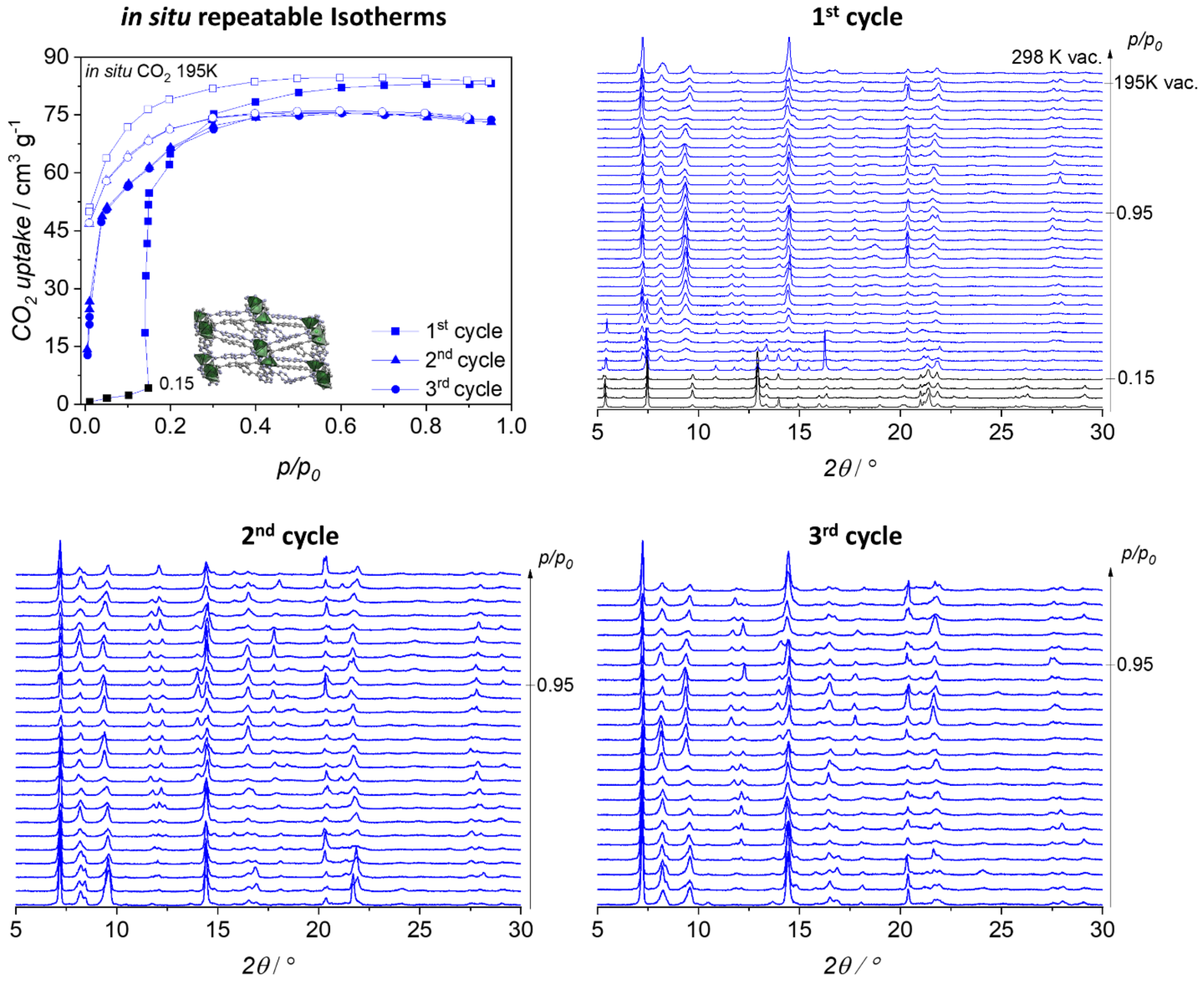

Figure S4 Three CO_2 adsorption (full symbols) and desorption (open symbols) cycles at 195 K juxtaposed with corresponding in situ PXRD patterns (λ = 1.540599 Å), measured in the almost all points of the presented isotherms.

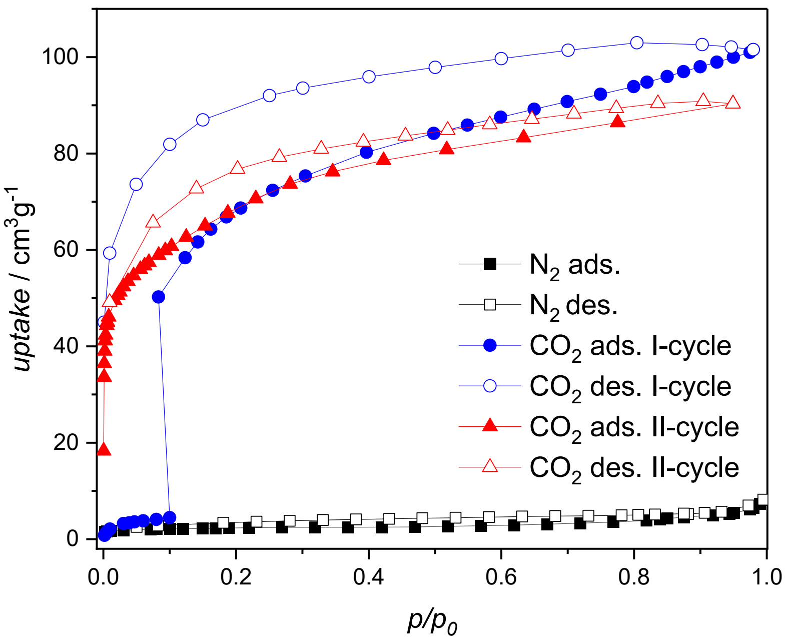

Figure S5 First indication of shape-memory effect: adsorption isotherms of CO_2 (195 K) and N_2 (77 K) for activated (exchange DMF to DMC, fine vacuum at 80 °C) Zn-terp-β $[Zn_2(nda)_2(terp)]_n$. Full symbols – adsorption; open symbols – desorption.

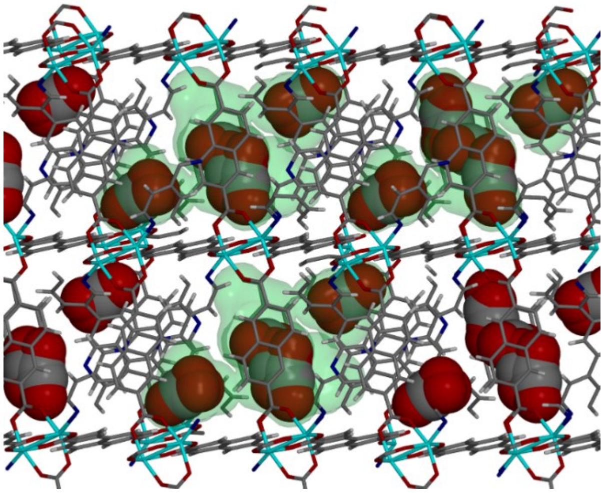

Figure S6 CO_2 binding sites resolved from *in situ* SC-XRD data collecting during the CO_2 adsorption at 296 K. The solvent accessible volume shown as green Connolly surfaces[14] was generated with a probe radius of 1.5 Å.

[14] Connolly, M. L. Solvent-Accessible Surfaces of Proteins and Nucleic Acids. Science, 1983, 221, 709.

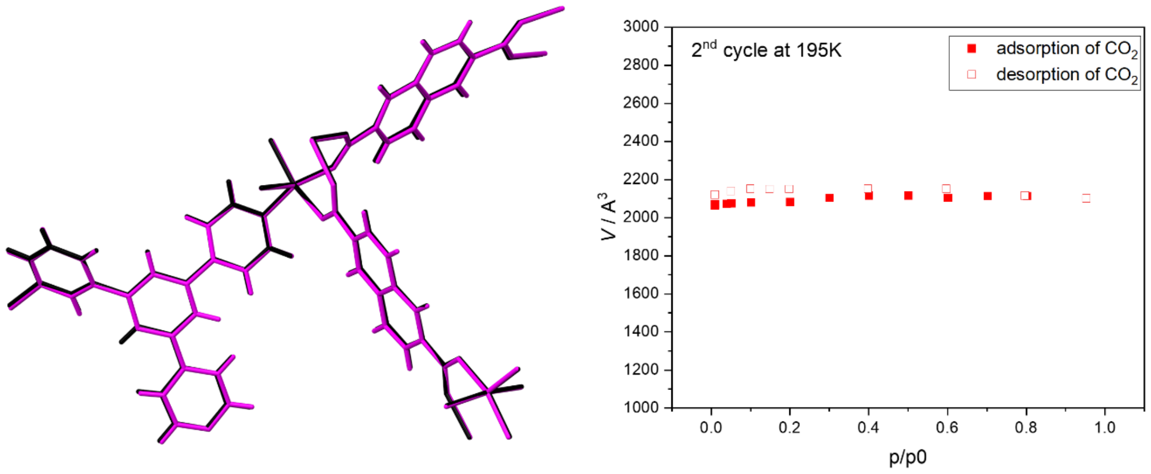

Figure S7 An overlay (left) of the shape-memory MOF structures under reduced (black) and 10 bar CO_2 (magenta) pressure juxtaposed with the unit cell volume changes during the 2nd adsorption cycle (see Fig. S4).

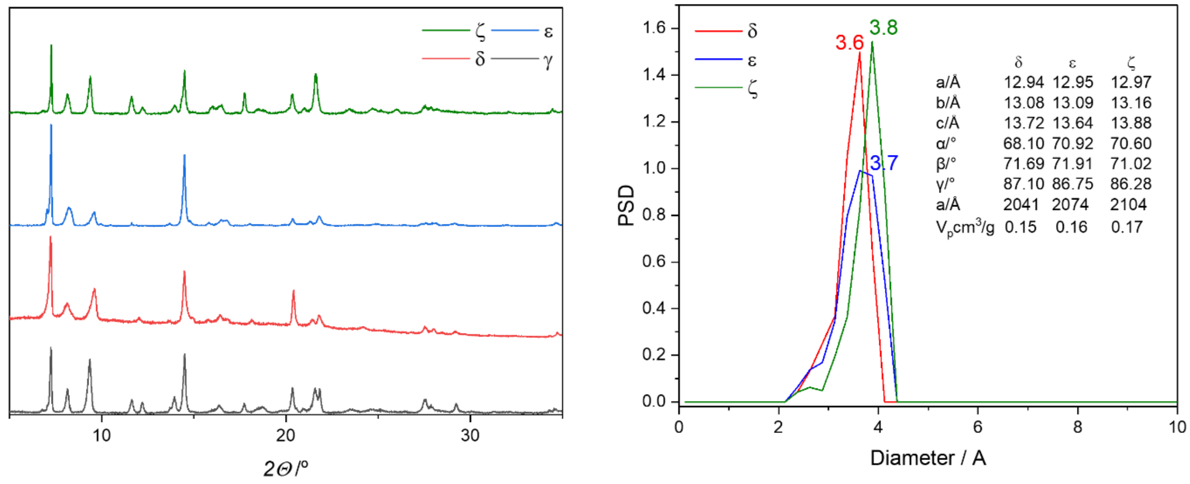

Figure S8 Experimental PXRD patterns of Zn-terp-γ, Zn-terp-δ, Zn-terp-ε and Zn-terp-ζ showing that all structure are analogous (left). Pore size distribution (PSD) for Zn-terp-δ, Zn-terp-ε and Zn-terp-ζ juxtaposed with unit cell parameters and theoretical pore volume calculated with Zeo++ software (right); all calculations were made with guest molecules excluded.

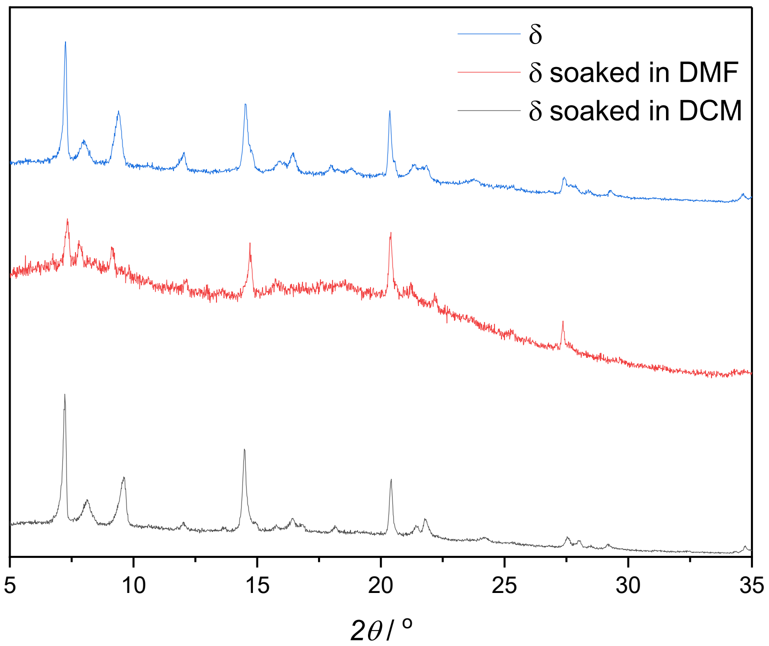

Figure S9 Comparison of the the PXRD patterns of as-synthesised δ-phase with those after soaking for 24h in DCM and DMF. Immersion of the α-phase in DMF significantly reduces crystallinity of the sample, while DCM has not impact on the sample.

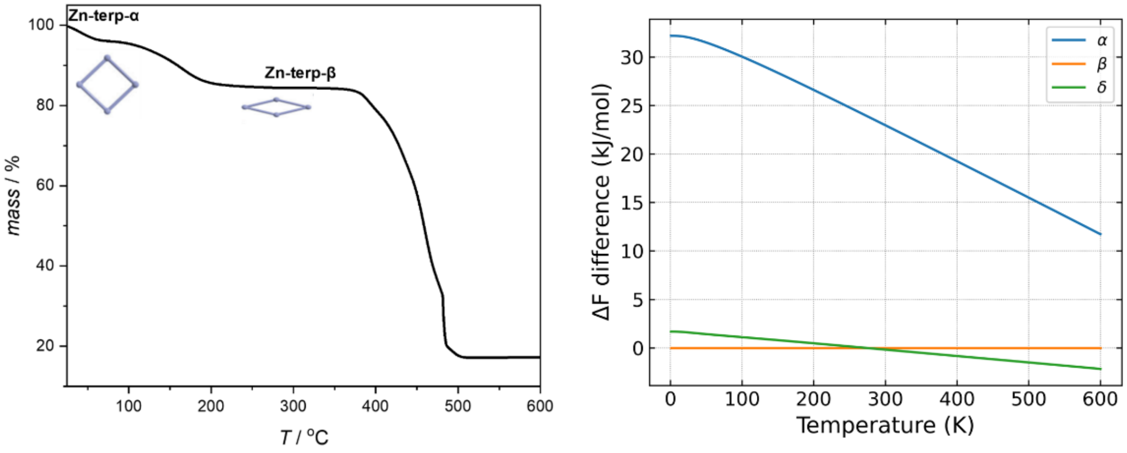

Figure S10 Thermal stability (left) of Zn-terp-α $[Zn_2(nda)_2(terp){\cdot}2DMF]_n$ indicating structural transformation into Zn-terp-β $[Zn_2(nda)_2(terp)]_n$ juxtaposed with calculated temperature-dependent relative free energy (right) of the α- ,β- ,δ- phases with free energy of β as a reference; Energy unit is given per mole of Zn paddle-wheel.

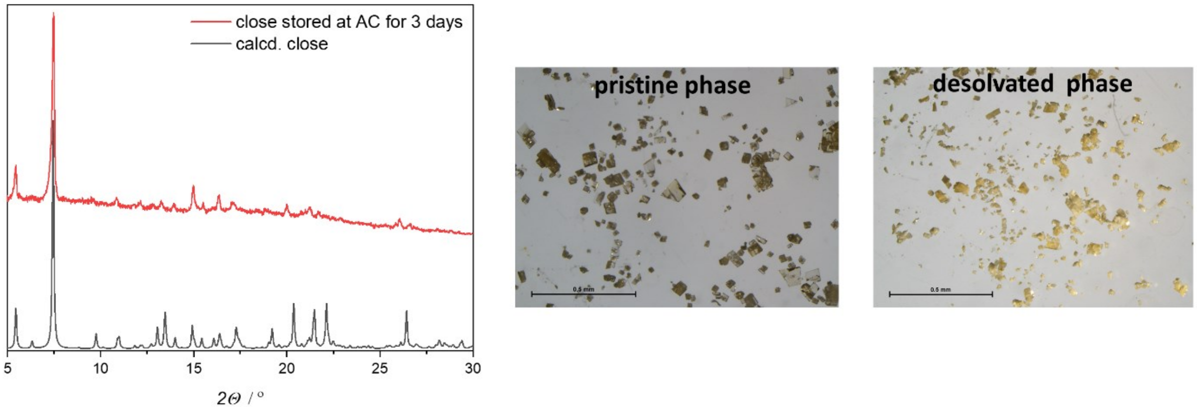

Figure S11 Experimental and calculated PXRD patterns of the desolvated materials Zn-terp-β (left) juxtaposed with optical image of it (right). AC - ambient conditions.

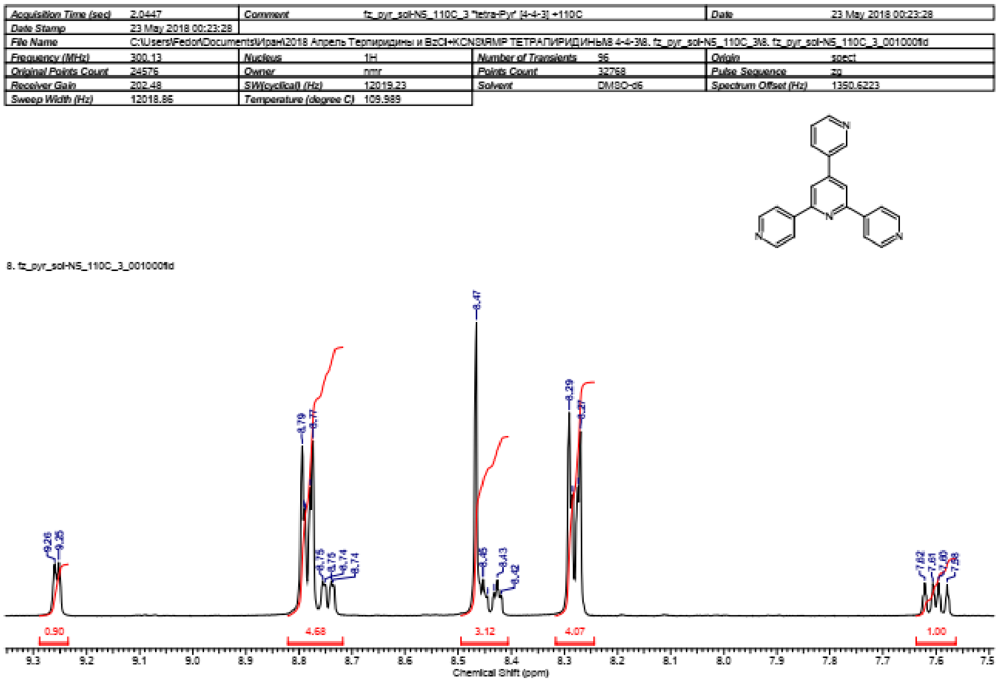

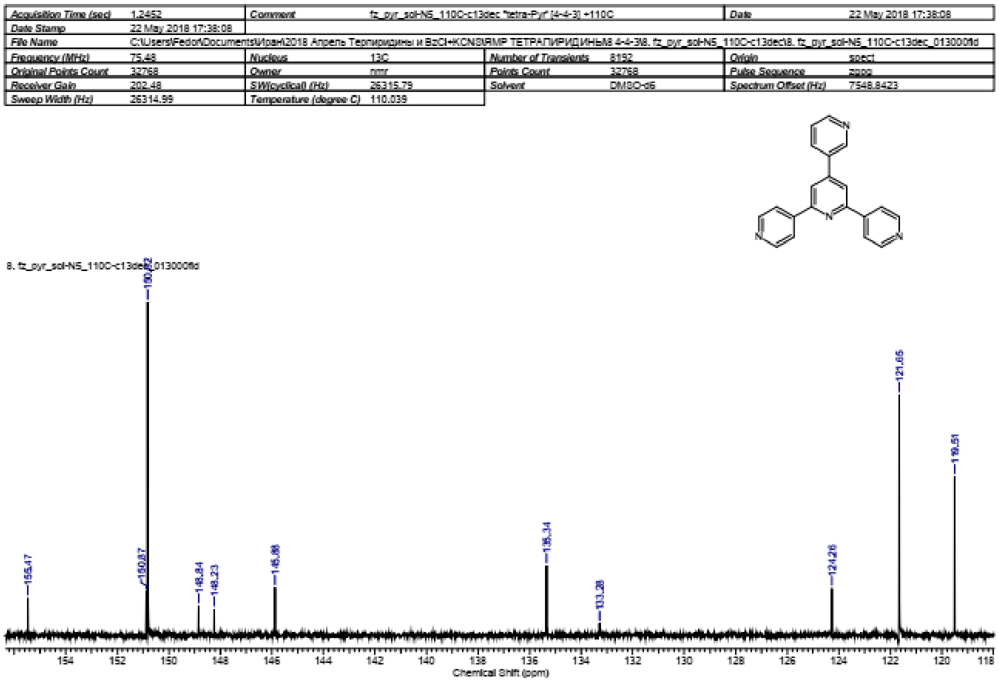

Figure S12 (top)^{1}H NMR spectrum of terp in DMSO-d6 and (down) ^{13}C NMR spectrum of terp in DMSO-d6.

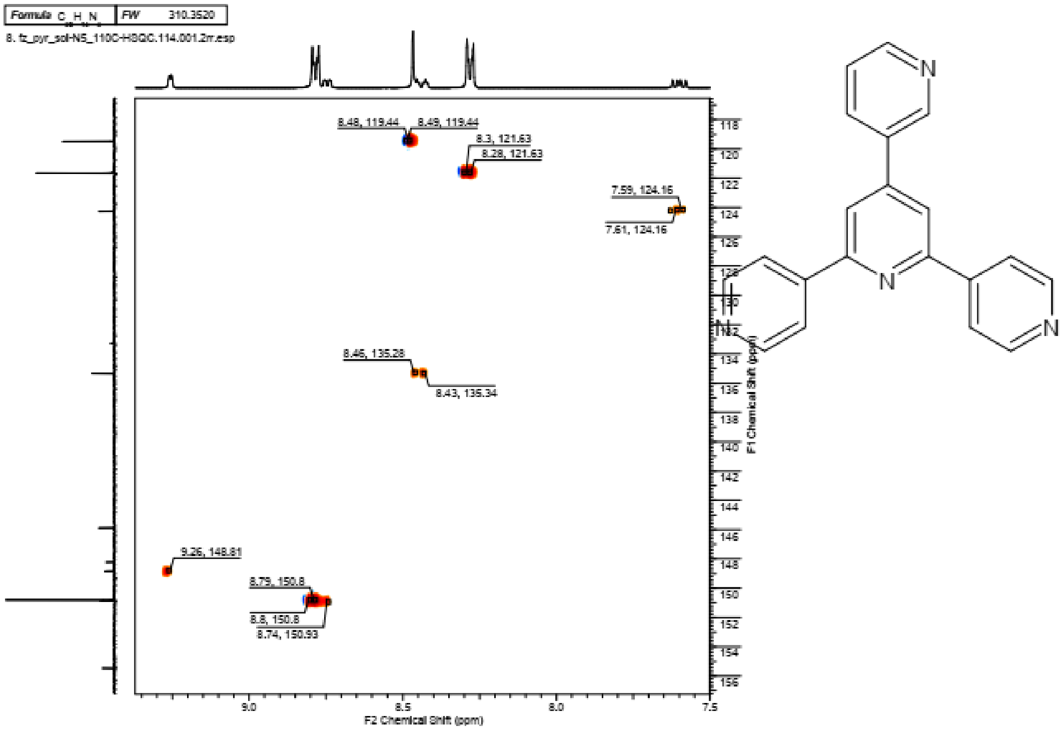

Figure S13 HSQC spectrum of terp.

Tables

Table S1 Crystallographic data for Zn-terp-α, β, δ, ε and ζ.

phase	**Zn-terp-α**	**Zn-terp- β**	**Zn-terp-δ**	**Zn-terp-ε**	**Zn-terp-ζ**
Empirical formula	$C_{88}H_{52}N_8O_{16}Zn_4$	$C_{44}H_{26}N_4O_8Zn_2$	$C_{44}H_{28}N_4O_{10}Zn_2$	$C_{44}H_{25}N_4O_8Zn_2$	$C_{45}H_{26}N_4O_{10}Zn_2$
Formula weight/ g/mol	1738.85	869.43	902.94	868.42	913.44
Temperature/K	298	100.15	100.03	296(2)	296(2)
Crystal system	triclinic	triclinic	triclinic	triclinic	triclinic
Space group	$P\bar{1}$	$P\bar{1}$	$P\bar{1}$	$P\bar{1}$	$P\bar{1}$
a/Å	13.0274(4)	9.513(3)	12.9487(17)	12.9510(10)	12.9725(8)
b/Å	13.1290(4)	16.732(3)	13.0756(17)	13.0850(9)	13.1154(7)
c/Å	33.0340(10)	24.136(3)	13.7254(18)	13.6422(11)	13.8810(9)
α/°	79.981(3)	92.745(13)	68.101(3)	70.922(7)	70.601(5)
β/°	88.137(2)	99.235(16)	71.685(3)	71.922(7)	71.022(6)
γ/°	84.517(3)	103.38(2)	87.109(3)	86.754(6)	86.280(5)
Volume/Å^3	5537.7(3)	3674.5(15)	2041.1(5)	2074.4(3)	2104.1(2)
Z	2	2	2	2	2
ρ_{calc}g/cm^3	1.043	1.572	1.469	1.390	1.442
μ/mm^{-1}	0.909	1.37	1.239	1.897	1.937
F(000)	1768	1768	919.0	882.0	928.0
Crystal size/mm^3	0.13 × 0.11 × 0.08	0.11 × 0.1 × 0.08	0.13 × 0.08 × 0.07	0.27 × 0.26 × 0.11	0.27 × 0.26 × 0.11
Radiation	MoKα (λ = 0.71073)	MoKα (λ = 0.71073)	MoKα (λ = 0.71073)	CuKα (λ = 1.54184)	CuKα (λ = 1.54184)
2Θ range for data collection/°	6.428 to 52.744	6.618 to 57.02	3.366 to 52.808	7.158 to 152.604	7.132 to 152.584
Index ranges	-16 ≤ h ≤ 16, -16 ≤ k ≤ 16, -41 ≤ l ≤ 41	-10 ≤ h ≤ 12, -20 ≤ k ≤ 20, -31 ≤ l ≤ 29	-14 ≤ h ≤ 16, -16 ≤ k ≤ 15, -16 ≤ l ≤ 17	-16 ≤ h ≤ 16, -16 ≤ k ≤ 14, -17 ≤ l ≤ 17	-15 ≤ h ≤ 16, -16 ≤ k ≤ 16, -17 ≤ l ≤ 17
Reflections collected	94077	37283	25930	16565	17521
Independent reflections	22595 [R_{int} = 0.1115, R_{sigma} = 0.1204]	15885 [R_{int} = 0.1430, R_{sigma} = 0.2602]	8331 [R_{int} = 0.1119, R_{sigma} = 0.1504]	8406 [R_{int} = 0.0503, R_{sigma} = 0.0659]	8585 [R_{int} = 0.0535, R_{sigma} = 0.0605]
Data/restraints/parameters	22595/99/1045	15885/2067/1045	8331/1236/635	8406/0/523	8585/14/584
Goodness-of-fit on F^2	0.96	1.028	1.062	1.039	1.032
Final R indexes [I>=2σ (I)]	R_1 = 0.0592, wR_2 = 0.1238	R_1 = 0.1219, wR_2 = 0.2950	R_1 = 0.0979, wR_2 = 0.1924	R_1 = 0.0827, wR_2 = 0.2226	R_1 = 0.0794, wR_2 = 0.2223
Final R indexes [all data]	R_1 = 0.1021, wR_2 = 0.1380	R_1 = 0.2493, wR_2 = 0.3644	R_1 = 0.1797, wR_2 = 0.2238	R_1 = 0.1151, wR_2 = 0.2558	R_1 = 0.1095, wR_2 = 0.2507
Largest diff. peak/hole / e Å^{-3}	0.38/-0.38	2.14/-1.41	0.82/-1.12	1.94/-0.87	1.39/-0.64

Table S2 Evolution of π-π interactions observed in different phases of Zn-terp-x.

	**l (Å)**	**θ (°)**	**r (Å)**
**Zn-terp-α**; $Zn_2(nda)_2(terp)\cdot 2DMF$			
PyN2….PyN7	4.506(6)	1.56	2.301(4)
PyN6…PyN2	4.346(5)	14.39	2.234(4)
**Zn-terp-β** $Zn_2(nda)_2(terp)$			
PyN4….PyN6	3.650(5)	14.05	1.408 (4)
PyN2…PyN7	3.516(5)	1.30	2.337(4)
PyN3…PyN5	3.993(4)	13.57	1.397(3)
**Zn-terp-δ**; $Zn_2(nda)_2(terp)\cdot 2H_2O$			
PyN2….PyN3*	3.596(6)	16.33	1.373(6)
PyN2*….PyN3	3.596(6)	14.39	1.373(6)
**Zn-terp-ε**; $Zn_2(nda)_2(terp)$			
PyN2….PyN3*	3.598(6)	16.53	1.373(6)
PyN2*….PyN3	3.598(6)	14.39	1.373(6)
**Zn-terp-ζ**; $Zn_2(nda)_2(terp)\cdot CO_2$			
PyN2….PyN3*	3.593(5)	16.33	1.383(6)
PyN2*….PyN3	3.593(5)	14.69	1.383(6)

l - the centroid–centroid distance, **θ** - interplanar angle, **r**- the offset of interacting rings.[15]

Table S3 Lattice parameters and relative energies of selected structures.

**Zn-terp-x**	**Experimental lattice parameters**	**Experimental volume (Å^3)**	**DFT lattice parameters**	**DFT volume (Å^3)**
**α**	13.0274 Å<br>13.1290 Å<br>33.0340 Å<br>78.98°<br>88.14°<br>84.52°	5537.68 Å^3	13.1219 Å<br>13.2397 Å<br>32.7290 Å<br>78.88°<br>88.53°<br>83.01°	5537.68 Å^3
**β**	9.5130 Å<br>16.7320 Å<br>24.1360 Å<br>92.75°<br>99.24°<br>103.38°	3674.55 Å^3	9.1964 Å<br>16.6580 Å<br>24.4220 Å<br>92.97°<br>99.58°<br>102.53°	3586.18 Å^3
**δ**	12.9487 Å<br>13.0756 Å<br>13.7254 Å<br>68.10°<br>71.69°<br>87.11°	2041.08 Å^3	13.0481 Å<br>13.1322 Å<br>12.8956 Å<br>70.25°<br>72.89°<br>87.28°	1984.35 Å^3

Table S4 Relative free energies for α and β phases with free energy of δ as a reference. Energies given per 1 Zn paddlewheel.

$\boldsymbol{T}$ **(K)**	$\boldsymbol{\Delta F_\alpha - \Delta F_\beta}$ **(kJ/mol)**	$\boldsymbol{\Delta F_\delta - \Delta F_\beta}$ **(kJ/mol)**
0 (electronic energy)	36.02	2.04
1	32.19	1.68
195	26.76	0.55
298	23.02	-0.09

[15] M.L. Główka, D. Martynowski, K. Kozłowska, *Journal of Molecular Structure*, 1999, 474, 81-89.
